# P-1174. MBI-CLABSI Cluster in Pediatric Oncology Patients with Hematologic Malignancies Introduces New Barrier in Fluoroquinolone Prophylaxis

**DOI:** 10.1093/ofid/ofae631.1360

**Published:** 2025-01-29

**Authors:** Karlye Jowers, Matt Mason, Margaret Gilman, Jennifer Vodzak

**Affiliations:** Nemours Children's Health, wilmington, Delaware; Nemours Children's Hospital, Delaware, Wilmington, Delaware; Nemours Children's Hospital, Delaware, Wilmington, Delaware; Nemours Children's Hospital, Wilmington, Delaware

## Abstract

**Background:**

Levofloxacin is preferred for prophylaxis during neutropenic periods in pediatric patients with high-risk acute myeloid and lymphoblastic leukemias (AML and ALL, respectively). The goal is to decrease risk of morbidity and mortality from bacteremia related to central venous lines (CVL) and chemotherapy-induced mucosal barrier injury (MBI), but antibacterial resistance may develop. Our institution observed an unexpected increase in MBI-central line associated bloodstream infections (MBI-CLABSI) in 2023, prompting a multidisciplinary review of the MBI-CLABSI cluster.

**Figure:**
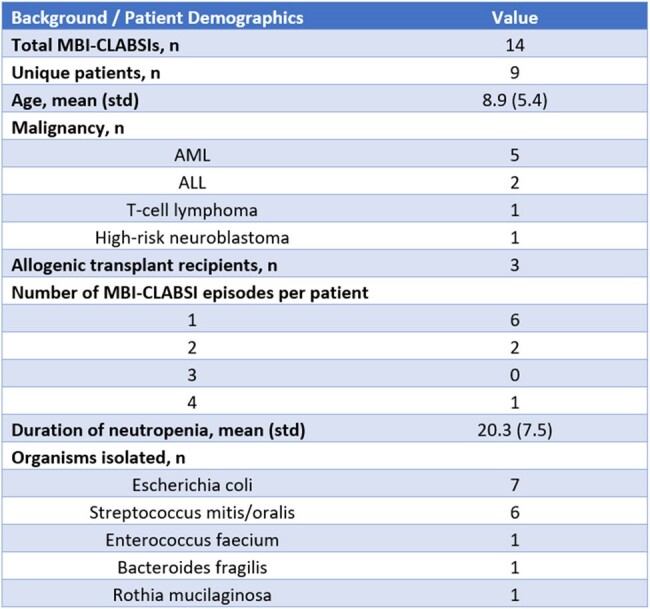

**Methods:**

Retrospective chart review was performed to characterize a cluster of MBI-CLABSI events occurring in pediatric patients with AML or ALL from 2020-2023 at our 220-bed, urban/suburban, free-standing children’s hospital. Descriptive analysis of the 2023 cluster events was performed, including patient demographics, diagnosis, transplant status, pathogen and prophylactic drug data to identify commonalities and potential harm-reduction interventions.

**Results:**

Rate of MBI-CLABSI were steady from 2020-2022 (0.24) and increased to 1.11 per 1,000 central line days in 2023 in patients with AML or ALL. Standard infection ratio of 3.69, p-value 0.0001. 85.7% (n=12/14) MBI-CLABI occurred in patients who had received prophylactic levofloxacin. Almost all organisms causing the MBI-CLABSIs were levofloxacin resistant (98%) and most (58%) were resistant to at least one additional antibiotic. Patients with multiple MBI-CLABSI events with a levofloxacin-resistant organism during the cluster year was not uncommon (66.7%; n=8/12)). . In this subset, patients continued to receive prophylactic levofloxacin despite having earlier infections caused by a levofloxacin-resistant pathogen.

**Conclusion:**

Use of levofloxacin prophylaxis for neutropenia episodes in high-risk AML and ALL patients may increase the risk of MBI-CLABSI events with levofloxacin-resistant organisms, thus reducing the effectiveness of the prophylaxis goal to prevent serious bacterial infections and reduce harm. Further studies are needed to better delineate optimal use of prophylactic antibiotics by incorporating population-level antibiograms and patient-specific infection histories.

**Disclosures:**

**All Authors**: No reported disclosures

